# Editorial: Human-robot interaction in industrial settings: new challenges and opportunities

**DOI:** 10.3389/frobt.2025.1652426

**Published:** 2025-08-05

**Authors:** Sara Bouraine, Mehdi Ammi, Kurt Geihs, Abdelfetah Hentout, Abderraouf Maoudj, Fouad Yacef

**Affiliations:** ^1^ Robotics and Industrial Automation, Center for Development of Advanced Technologies (CDTA), Algiers, Algeria; ^2^ SAMOVAR Telecom SudParis, CNRS, Institut Polytechnique de Paris, Paris, France; ^3^ Scientific Center for Information Systems Design (ITeG), University of Kassel, Kassel, Germany; ^4^ National Higher School of Advanced Technologies (ENSTA), Algiers, Algeria; ^5^ SDU Biorobotics, Maersk Mc-Kinney Moller Institute, University of Southern Denmark, Odense, Denmark

**Keywords:** human-robot interaction, human-robot collaboration, safe interactions, context-aware decision-making, industrial settings

## Background

Industrial settings are undergoing a progressive transformation toward collaborative work environments, where humans and robots share workspaces and collaborate closely. This shift has brought Human-Robot Interaction (HRI) to the center of investigation and improvement in such settings (Weiss et al., 2021; Kopp et al., 2021; Rodriguez-Guerra et al., 2021; Baltrusch et al., 2022; Wei et al., 2023; Othman and Yang, 2023). HRI encompasses a range of interaction levels, including coexistence, cooperation, and collaboration, depending on the manner humans and robots interact to accomplish shared tasks (Hentout et al., 2019). The objective of this study is to combine human cognitive flexibility and decision-making expertise with robotic precision and strength to enhance productivity and improve the wellbeing of workers. Nevertheless, this combination presents numerous challenges, particularly in scenarios involving full-autonomous robots operating directly alongside humans, as opposed to conventional robots that are confined into safety cages. The main issues in such conditions include ensuring natural and safe interaction Maurtua et al. (2017); Robla-Gómez et al. (2017); Samarathunga et al. (2025), predicting human intentions Liu et al. (2019); Orefice et al. (2016), learning from human behaviors Mukherjee et al. (2022), efficient tasks planning Noormohammadi-Asl et al. (2025), managing hardware and communication failures Honig and Oron-Gilad (2018), guaranteeing intelligent and context-aware decision-making Quintas et al. (2018), and robust navigation in real-world environments (Bouraine et al., 2023; Loganathan and Ahmad, 2023). The present research area focuses on creating effective, safe, and trustworthy HRI industrial systems.

## Scanning the Research Topic

This Research Topic brings together a Research Topic of five groundbreaking articles presenting the latest advances in the HRI field. The first article Wolffgramm et al. bridges the gap between human-cobot interaction and work perceptions in production units. This research explores an unaddressed issue by studying how operators use job decision latitudes to design synergistic human-cobot interdependencies in such an application and to evaluate if the operator is both willing and able to use the decision latitudes related to cobot tasks. Job decision latitude refers to “the discretion permitted to the worker in deciding how to meet work demands” (Karasek, 1979, p. 285). In the context of human-cobot collaboration, it reflects the operator’s ability to adapt and change their interactions with the cobot before, during, and after task execution. To investigate this phenomenon, researchers built one manual and three human-cobot production units with varying operator autonomy levels. These units were utilized in an assembly simulation that involved 40 students. The findings indicated that the productivity of human-cobot production units is strongly improved with greater job decision latitude. Nevertheless, the utilization of these decision latitudes is predominantly oriented towards the objective of reducing time, rather than paying attention to interaction quality or fostering sustainable work practices. From this vantage point, it is evident that operators tend to employ decision latitude to augment cobot velocity or to reallocate tasks in accordance with the execution speed. The research underscores the pivotal role of instrumental assistance and operator behavior in establishing effective human-cobot collaboration and safer psychological conditions, thus providing a robust foundation for future research in modern production environments.

Since 1940s, nuclear industry uses teleoperation and robotic systems to perform tasks within hazardous and confined environments. Moreover, manipulators played a crucial role in handling dangerous substances and operating in inaccessible or unsafe areas. Additionally, technological progress has expanded the capabilities and missions range that these systems are able to carry out. Due to extremely challenging environmental constraints, outdated structural data, and limited visibility, remote inspection and handling are frequently the only solution in nuclear decommissioning. The execution of such tasks necessitates extensive training and detailed planning. There is also a growing need to speed up deployment and increase activity volumes, all the while maintaining rigorous safety and performance standards. New robotic technologies, including Haptic Digital Twins and semi-autonomous control systems, have yielded significant improvements in environmental feedback. The implementation of these advanced technologies has the potential to significantly improve the efficiency of nuclear decommissioning processes. The second article Lopez Pulgarin et al. discusses latest industry best practices for teleoperation and outlines the manner in which such innovations could further improve efficiency, safety, and operational effectiveness. These advances could play a key role in improving nuclear decommissioning operations.

The utilization of social robots in product advertising is gaining prominence, and their potential impact on sales is a subject of interest. In Mizuho et al., the authors conducted a study in a grocery store to examine the manner customers remember advertised products. The study compared the impact of physical robots and virtual agents. According to the researchers, there is a possibility that customers would better remember content when hearing it from multiple agents. Further, the physical robots would elicit more favorable social feedback and remember them better than humans. However, the results did not support these assumptions, and there were no significant variations in memory based on agent type or number. Furthermore, researchers found that when people interacted with a physical robot, they perceived well the social presence, but not when they used virtual agents. This implies that the agent type does not affect memory, but the social interaction with a physical robot may further enhance it. The study provides valuable insights for future studies on the way different agent types affect customer involvement and retention in advertising.

Authors in Venås et al. investigated how quickly and successfully users can learn to perform a cooperative lifting task with a cobot. A total of 32 adults between the ages of 20 and 54 participated in the study. The experimental setup involved a gamified training setup, wherein the subjects interacted with a robot under three distinct role conditions: human-led, robot-led, and shared leadership. The user movement was tracked using Inertial Measurement Units (IMU) sensors. The findings indicated that all participants, irrespective of age, gender, job type, gaming experience, or familiarity with robots, accomplished successful cooperation within seven or fewer attempts. Whereas, a few user background factors, such as occupation and gaming habits, may influence learning speed, the overall learning progression was strong across the board. The outcomes suggested that Human-Robot Collaboration (HRC) can be effectively adopted in industry by a different range of users with minimal training, thereby highlighting its practical potential for broader deployment.

In Zakaria et al., the authors presented a novel decision-making framework for HRC that improves adaptability and flexibility compared to existing strategies. Traditional HRC approaches emphasized task completion, treating it as an optimization issue with fixed reward capacities. This made it difficult to alter performance metrics within the same system. The proposed system separates task completion (as a constraint) from collaboration execution (as a modifiable reward), allowing simple adjustment of performance criteria between scenarios. This structure gave superior control over interaction dynamics and ensured real-time adjustment of robot behavior to human activities. The decision-making process is based on game theory, particularly utilizing Nash Equilibrium and perfect-information extensive form. This empowers the framework to handle different execution objectives, such as optimizing task time, while considering the probability of human errors. The approach was validated through simulations and a real-assembly task utilizing a construction kit, thereby demonstrating its efficacy and adaptability in dynamic collaborative environments.

## Conclusion

This Research Topic brings together state-of-the-art contributions within the field of HRI in industrial settings, with the objective of serving as a substantial resource for researchers in the upcoming years. It simultaneously underscores the manifold issues and future perspectives that remain open and require further exploration.
